# Albumin nanoparticles increase the anticancer efficacy of albendazole in ovarian cancer xenograft model

**DOI:** 10.1186/s12951-015-0082-8

**Published:** 2015-03-25

**Authors:** Lubna Noorani, Martina Stenzel, Roger Liang, Mohammad H Pourgholami, David L Morris

**Affiliations:** Centre for Advanced Macromolecular Design (CAMD), School of Chemistry, University of New South Wales, Sydney, NSW Australia; Department of Surgery, St. George Clinical School, Faculty of Medicine, University of New South Wales, Kogarah, NSW Australia; School of Biomedical Sciences and Pharmacy, University of Newcastle, Newcastle, NSW Australia

**Keywords:** Angiogenesis, Albendazole, BSA-ABZ 10 nm, Nab-ABZ 200 nm, VEGF, Ascites, Ovarian cancer

## Abstract

**Background:**

The poor prognosis of patients with drug resistant ovarian cancer and the lack of targeted therapy have raised the need for alternative treatments. Albendazole (ABZ) is an anti-parasite compound capable of impairing microtubule formation. We hypothesized that ABZ could be repurposed as a potential anti-angiogenic drug due to its potent inhibition of vascular endothelial growth factor (VEGF) in ovarian cancer with ascites. However, the poor aqueous solubility of ABZ limits its potential for cancer therapy. In this study, we have assembled ABZ with bovine serum albumin into nanoparticles with a size range of 7–10 nm (BSA-ABZ) and 200–250 nm (Nab-ABZ). We further examined the anticancer effects of ABZ carrying nanoparticles in ovarian cancer cells, in both *in vitro* and *in vivo* models.

**Results:**

Drug release studies demonstrated that about 93% of ABZ was released from BSA-ABZ 10 nm in comparison to 83% from Nab-ABZ 200 nm at pH 7.4 in 8 days. *In vitro* cell proliferation studies showed that the BSA-ABZ 10 nm exhibited the highest killing efficacy of ovarian cancer cells with surprisingly least toxicity to healthy ovarian epithelial cells. Confocal microscopy and fluorescence activated cell sorting analysis (FACS) revealed more efficient internalization of the BSA-ABZ 10 nm by cancer cells. For *in vivo* studies, we examined the tumor growth, ascites formation and the expression of VEGF and secreted protein acidic and rich in cysteine (SPARC) in tumor samples and only VEGF in plasma samples. The BSA-ABZ 10 nm reduced the tumor burden significantly (p < 0.02) at a much lower drug dose (10 μg/ml) compare to free drug. Both formulations were capable of suppressing the ascites volume significantly (p < 0.05) and reducing the number of ascites cells. The expression of VEGF and SPARC was also reduced, which indicates the underlying therapeutic mechanism of the ABZ.

**Conclusion:**

Our data suggest that the BSA-ABZ may hold promise for the treatment and control of progression of ovarian cancer with ascites. However further studies are required to examine the efficacy of both the formulations in aggressive models of recurrent ovarian cancer with respect to particle size and dosing parameters.

**Electronic supplementary material:**

The online version of this article (doi:10.1186/s12951-015-0082-8) contains supplementary material, which is available to authorized users.

## Background

Angiogenesis, the development of new capillaries from existing vasculature, plays a key role in the pathogenesis of metastases [1]. VEGF is important in tumor angiogenesis, growth and metastasis [2,3], especially in ovarian cancers and other solid tumors [4,5]. Substantial evidence suggests that VEGF promotes the formation of ascites and is present at a very high levels in ascites of patients with advanced ovarian cancer [6]. Patients suffering from recurrent ovarian cancer are known to be affected by malignant ascites (accumulation of fluid containing cancer cells in the abdomen) and develop intraperitoneal metastases, thus leading to treatment failure. Current therapies that exist for treating ascites are salt restriction, diuretics, radioactive isotopes, paracentesis and shunt placement [7]. Recently, the anti-angiogenic agents, such as Bevacizumab and the VEGF trap, have been shown to be effective in the reduction of ascites [8]. VEGF trap had been combined with Paclitaxel (PTX) to inhibit tumor and ascites and prolong patient’s survival [9]. It has been shown that the combination of VEGF inhibitor with chemotherapeutic agents can significantly inhibit tumor growth and metastasis and suppress the multidrug resistance (MDR) gene in the tumor endothelium [10].

Albendazole (ABZ), Methyl [5-(Propylthio)-1-H-Benzimidazol-2Yl] Carbamate, is an anti-anthelminthic drug and has been explored as a potential inhibitor of VEGF [11], hypoxia inducible factor 1-α [12] and tumor angiogenesis [13] over the past few years. The antitumor effect of ABZ is related to its inhibition of tubulin polymerization and G2 M phase arrest of cell cycle [14,15]. In combination with chemotherapeutics, ABZ shows superior anti-VEGF activity in a xenograft model of ovarian cancer [16]. ABZ also improves the antitumor activity in combination with tubulin binding agent 2- Methoxy estradiol [17]. In light of its anticancer properties coupled with VEGF inhibition, we hypothesized that ABZ could be repurposed as a new treatment of ovarian cancer.

ABZ has very low aqueous solubility (0.55 μg/ml) and bioavailability, which limits the potential use of ABZ in cancer treatment. We perceived that the use of nanoparticle albumin bound (nab) technology in the drug delivery system for ABZ may increase its solubility with selective delivery to the tumors. Nab-technology is basically non-covalent association of hydrophobic drugs with albumin and thus formation of nanoparticles that are readily dispersible in water without any solvent or surfactant. One of the most important features of the albumin based drug delivery system is the enhanced drug accumulation in tumor tissues due to the leaky vasculature of the angiogenic vessels in solid tumor [18]. The nab-based drug delivery system can also improve drug uptake by exploiting the biological pathways of albumin [19]. Abraxane® (nab-paclitaxel) was the first nab-based oncology product approved for human use by the FDA in 2005 [20,21]. Based on the concept of albumin bound Paclitaxel (Abraxane®), we aimed to develop a novel formulation with nanoparticle albumin bound ABZ.

The current study aimed at exploring nab-technology to solve the solubility problem of albendazole and reduce side effects by targeting tumor passively and increase the efficacy. The study also focused on to pursue a nano-sized drug delivery system (<50 nm) for better absorption in the peritoneal cavity and compared the effect of particle sizes in *in-vitro* and *in-vivo* models of ovarian cancer.

To achieve these, nanoparticles were prepared by optimizing drug to protein ratios in order to make formulation of 10 nm BSA-ABZ and 200 nm Nab-ABZ nanoparticles. Both formulations were examined for cellular uptake and cytotoxicity on ovarian cancer cells (OVCAR3 and SKOV3) and a human ovarian surface epithelial cell line (HOSE). Subsequently, we established an OVCAR3 xenograft tumor model to evaluate the antitumor effects of Nab-ABZ in *in vivo*. The *in-vivo* studies were particularly focused on the measurement of tumor burden, ascites fluid volume, number of ascites cells, VEGF and SPARC expression in tumor and VEGF concentration in plasma. Finally, the objective of this study was to determine the most effective formulation/particle size that may provide a rationale to select the most suitable regimens for subsequent pre-clinical and clinical evaluations, hopefully in the near future.

## Results

### Preparation, characterization and drug release

**Figure 1:**
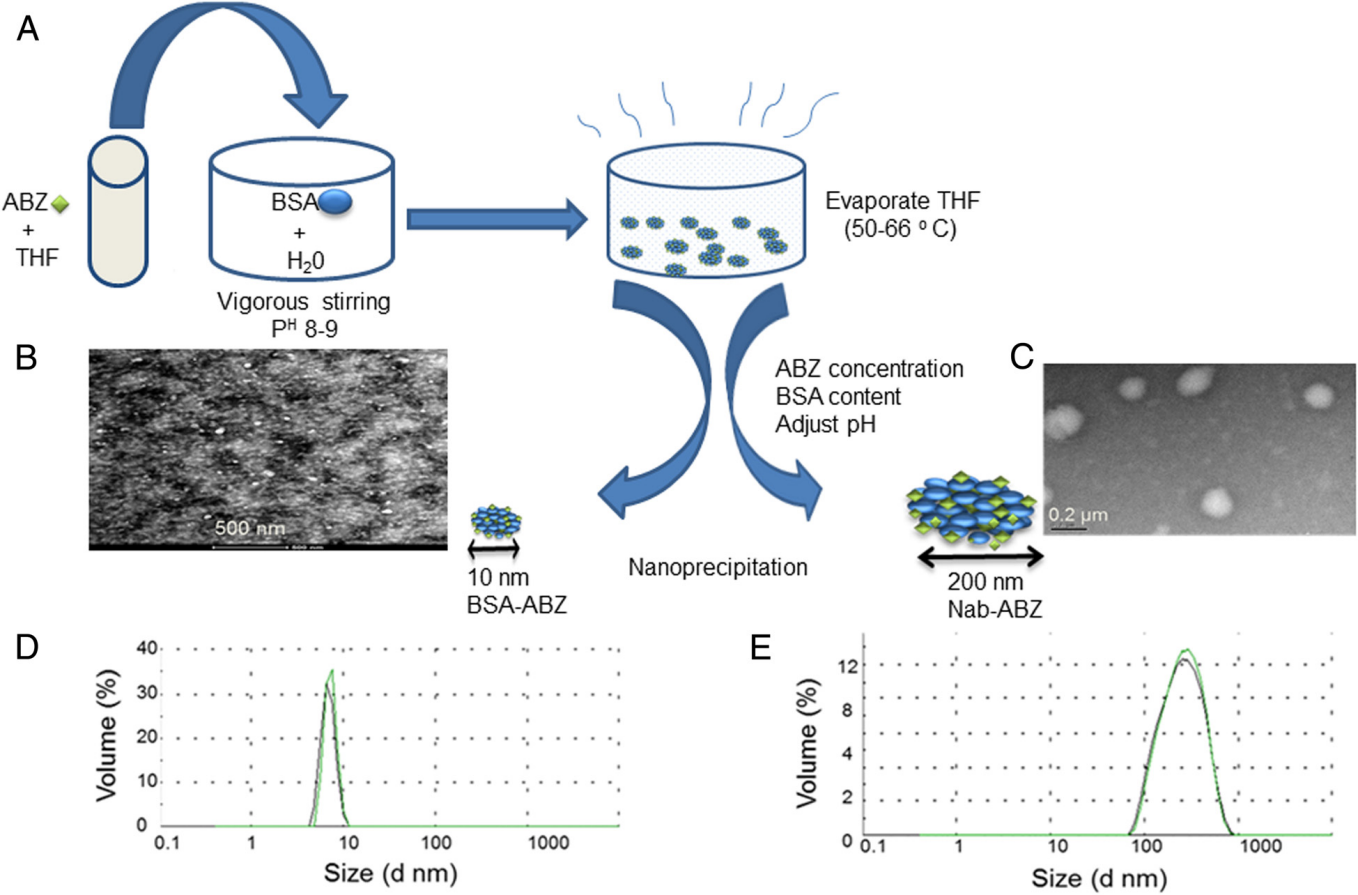
**Synthesis and characterization of nab-ABZ.** Schematic illustration of albendazole encapsulation by albumin nanoparticles **(A)**. TEM images of BSA-ABZ 10 nm **(B)**, the scale bar is 500 nm and TEM images of Nab-ABZ 200 nm **(C)**, and the scale bar is 0.2 μm. Size distribution (volume %) measured by DLS of BSA-ABZ 10 nm **(D)** and Nab-ABZ 200 nm **(E)**. Data are presented as the average of two measurements. TEM images confirmed the particle size of DLS.

**Figure 2:**
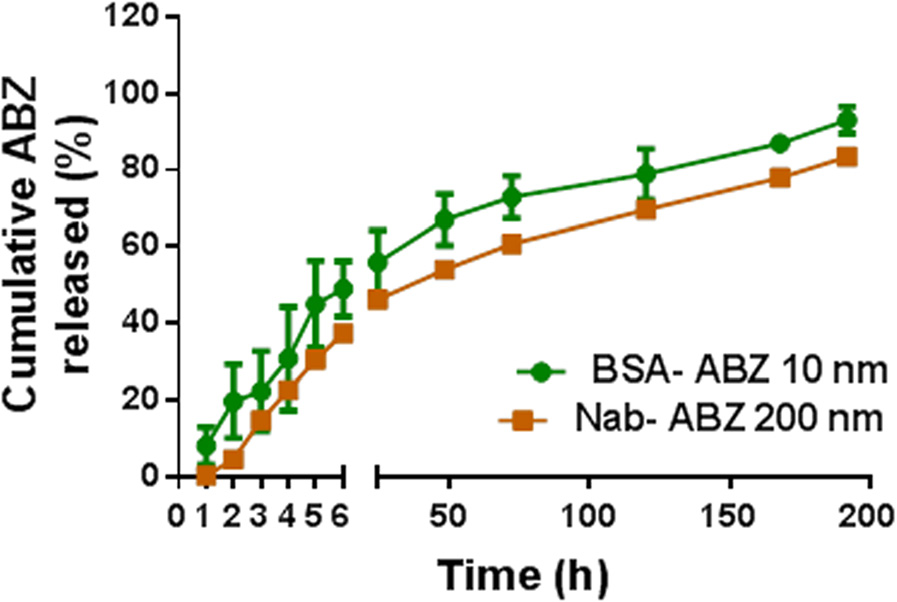
**Release profile of albendazole from albumin nanoparticles.** *In-vitro* release behaviour of ABZ from nanoparticle formulations BSA-ABZ 10 nm and Nab-ABZ 200 nm in PBS at pH 7.4. The nanoparticle dispersion was placed in an orbital shaker and shaken at 100 rpm at 37°C for 192 hours. The released ABZ concentration was measured by HPLC. Data are presented as mean ± SD (n = 2).

Drug loaded nanoparticles were prepared by a modification of the desolvation method as shown in Figure 1A. ABZ was dissolved in tetrahydrofuran (THF) and subsequently mixed with an aqueous solution of albumin under stirring. The mixture was finally heated up to the boiling point of THF to remove the solvent from final preparation. The formulations were prepared with a low ABZ to protein ratio (1:100) for 10 nm nanoparticles and (1:5) for 200 nm nanoparticles. Therefore, these formulations do not require any sedimentation by centrifugation to isolate aggregated particles (reaction products) and then subsequently evade washing steps to achieve 100% drug added in the formulation. Nab-ABZ with a particle size of 200 nm was synthesized using the same method with a higher concentration of ABZ in the initial solution. The nanoparticles were spherical in shape and possessed a smooth surface morphology as shown by transmission electron microscope (TEM) (Figure 1B and C). Dynamic light scattering (DLS) measurements demonstrated mono-modal particle size distribution for both nanoparticles (Figure 1D and see Additional file 1). Considering the size of BSA of around 5 nm, these results suggest the aggregation of only a few protein molecules in the case of the 10 nm particle. The change of the nanoparticle synthesis method may change the characteristics of particles [22]. However, there was no significant difference in release behaviour between the two formulations (Figure 2). 50% and 37% of the drug was released from BSA-ABZ 10 nm and Nab-ABZ 200 nm respectively in the first 6 hours at pH 7.4. The BSA-ABZ 10 nm reached 93% of accumulated release after 8 days in comparison to 83% for Nab-ABZ 200 nm. Overall, our release data showed an initial burst release followed by a steady release of ABZ for approximately 8 days, indicating that the delivery system was effective for an extended period of time.

### *In-vitro* cytotoxicity

**Figure 3:**
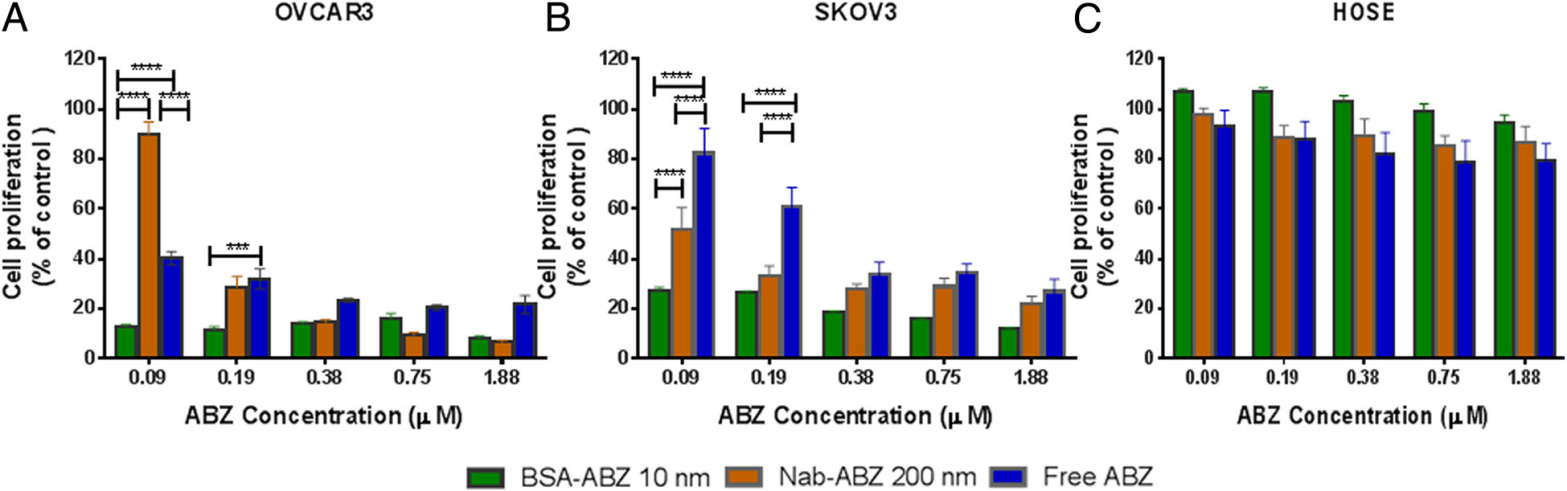
**Cytotoxicity of albendazole loaded albumin nanoparticles.** Comparison of the cytotoxic effects of BSA-ABZ 10 nm, Nab-ABZ 200 nm and free ABZ in ovarian cancer cells, OVCAR3 **(A)** SKOV3 **(B)** and HOSE **(C)** at different concentration of ABZ (μM) for 72 hours. Each experiment was conducted three times with replicates of 4 to 8 for each drug concentration. Data are presented as mean ± SD (n = 3).

We assessed the cytotoxicity of BSA-ABZ 10 nm and Nab-ABZ 200 nm and free ABZ against ovarian cancer cells (OVCAR3 and SKOV3) and a normal ovarian cell line (HOSE) in *in vitro* culture for 72 hours. As depicted in Figure 3, nab-ABZ 200 nm and free ABZ exhibited a dose dependent cytotoxicity against ovarian cancer cells (Figure 3A and B). The BSA-ABZ 10 nm at the lowest concentration (0.09 μM) significantly inhibited cell proliferation compare to free ABZ and nab-ABZ 200 nm in both cell lines (OVCAR3 and SKOV3). Interestingly, no apparent toxicity was observed in HOSE cell line even at highest dose (1.88 μM). Nearly 20% cell’s proliferation inhibition was detected by free ABZ at the highest concentration in HOSE. Therefore, both formulations were toxic to the ovarian cells whereas nontoxic to the HOSE cell which indicated the possibility of targeted therapeutic effect against cancer (Figure 3C).

### Cellular Internalization

**Figure 4:**
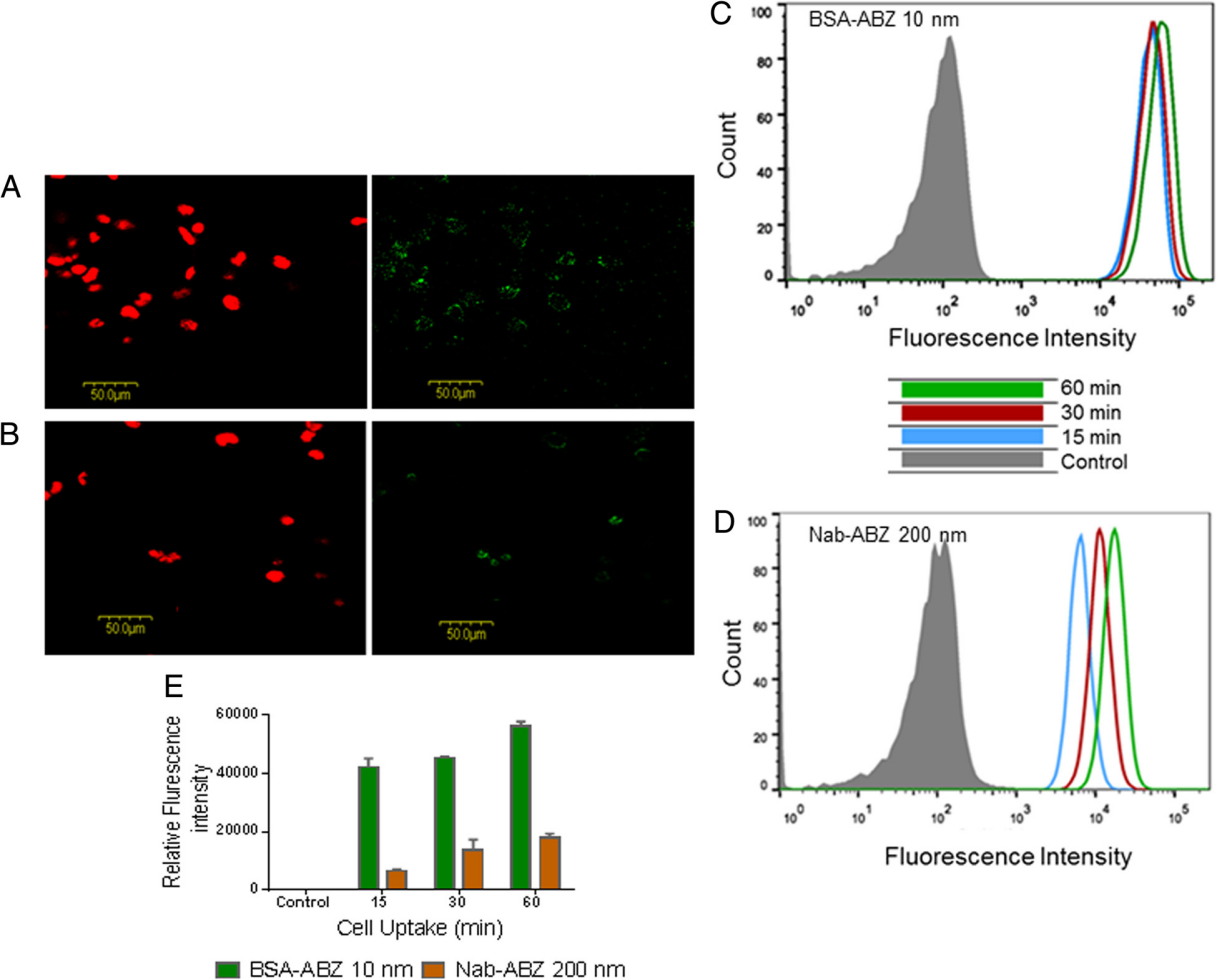
**Internalization of albumin nanoparticles.** Internalization of albumin nanoparticles was examined by confocal laser scanning microscope. SKOV3 cells were incubated with alexa-488 conjugated BSA-ABZ 10 nm **(A)** and Nab-ABZ 200 nm **(B)** and the nucleus was stained with PI (red). The fluorescence intensity and the cell images were obtained using two channels: green λ_ex/_ λ_em_ = 488 nm/ 492–508 nm for alexa-488 conjugated BSA NPs and red ( λ_ex/_ λ_em =_ 568 nm/ 612–622 nm) for the PI. Scale bar is 50 μm and magnification 60Χ with oil immersion lenses. Cells were treated with fluorescent nanoparticles for 15, 30 and 60 min and uptake of BSA-ABZ 10 nm **(C)** and Nab-ABZ 200 nm **(D)** was quantitatively measured by FACS analysis. Right shift of the chromatogram indicates more cellular uptake **(C)**. Relative fluorescence intensity was also measured by FACS analysis **(E)**. Data are presented as mean ± SD (n = 2).

The internalization of BSA-ABZ and Nab-ABZ in SKOV3 cells was visualized by confocal microscopy and the intensity of the fluorescent particles was measured by FACS analysis. The internalization of particles was depicted in Figure 4A and B for 10 nm and 200 nm respectively. The fluorescent intensity was significantly higher in the formulation containing Nab-ABZ 10 nm compared to 200 nm. Similarly, the uptake of BSA-ABZ 10 nm by SKOV3 cells was more prominent than that of Nab-ABZ 200 nm as measured by FACS analysis. In fact, there was no significant increase of fluorescence after 15 minutes incubation suggesting that most of the particles were taken up within 15 minutes (Figure 4C). Relative fluorescence intensity was detected at much higher levels in cells incubated with BSA-ABZ 10 nm compared to Nab-ABZ 200 nm (Figure 4E). Both FACS and microscopic observations confirmed that higher fluorescence intensity was detected in the cells incubated with BSA-ABZ 10 nm compared to Nab-ABZ 200 nm.

### *In-vivo* antitumor effect of ABZ nanoformulations

**Figure 5:**
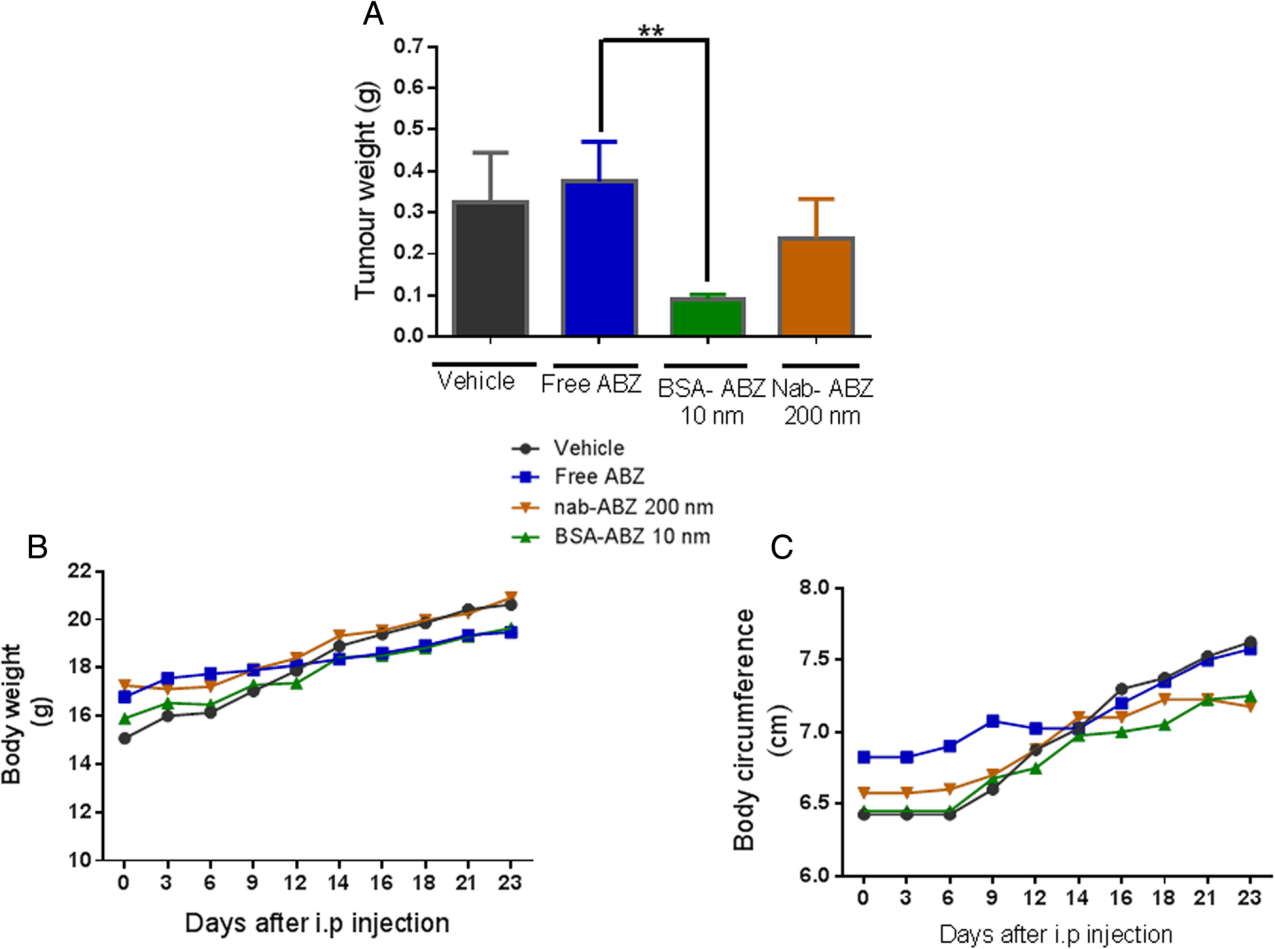
**ABZ nanoformulations lead to enhanced antitumor efficacy in the BALB/c nude mice bearing OVCAR3.** Treatment started at day 8 after intraperitoneal injection of OVCAR3 tumor cells at day 0. Vehicle, free ABZ, BSA-ABZ 10 nm and Nab-ABZ 200 nm nanoparticle formulations were administered on day 8, 11, 14, 17, 20, 23 by intraperitoneal injection in the mice. To compare the fate of nanoparticles *in-vivo*, different sizes of particles were administered regardless of particle concentration. **A)** Tumor weight average **B)** mice body weight **C)** mice body circumference (Mean ± SD, n = 4). Compared to free ABZ, no obvious toxicity observed to the mice which were demonstrated well maintained body weight of the mice. Three weeks following treatment BSA-ABZ 10 nm animals group reduced tumor burden very significantly (**p < 0.01) and revealed no evidence of disease, while animals treated with vehicle and free ABZ demonstrated extensive tumor burden. Body weight and abdominal circumference were measured twice weekly.

Antitumor efficacy of nanoparticle formulations was evaluated in a study using the human OVCAR3 ovarian cancer xenograft model. The mice were treated intraperitoneally with vehicle, free ABZ (50 mg/kg), BSA-ABZ 10 nm where ABZ concentration 10 μg/ml (0.5 mg/kg) and Nab-ABZ 200 nm where ABZ concentration 200 μg/ml (10 mg/kg). To assure accurate comparison of tumor burdens, all groups were sacrificed when control group developed clinical evidence of disease progression. Intraperitoneal administration of BSA-ABZ 10 nm, three times weekly for 3 weeks led to a significant reduction (p < 0.02) in average tumor weight compare to free ABZ (Figure 5A). In contrast, the tumor weight was not reduced to the same extent when using Nab-ABZ 200 nm group (Figure 5A). During the experiment, relative body weight and body circumference were also determined with the change of body weight as an indicator of toxicity (Figure 5B and C). None of animals treated with nanoparticles displayed evidence of major systemic toxicities including weight loss, inability to obtain food or water, hunched appearance, intra-abdominal adhesion or obstruction.

### ABZ nanoformulation reduces ascites fluid in peritoneal cavity

**Figure 6:**
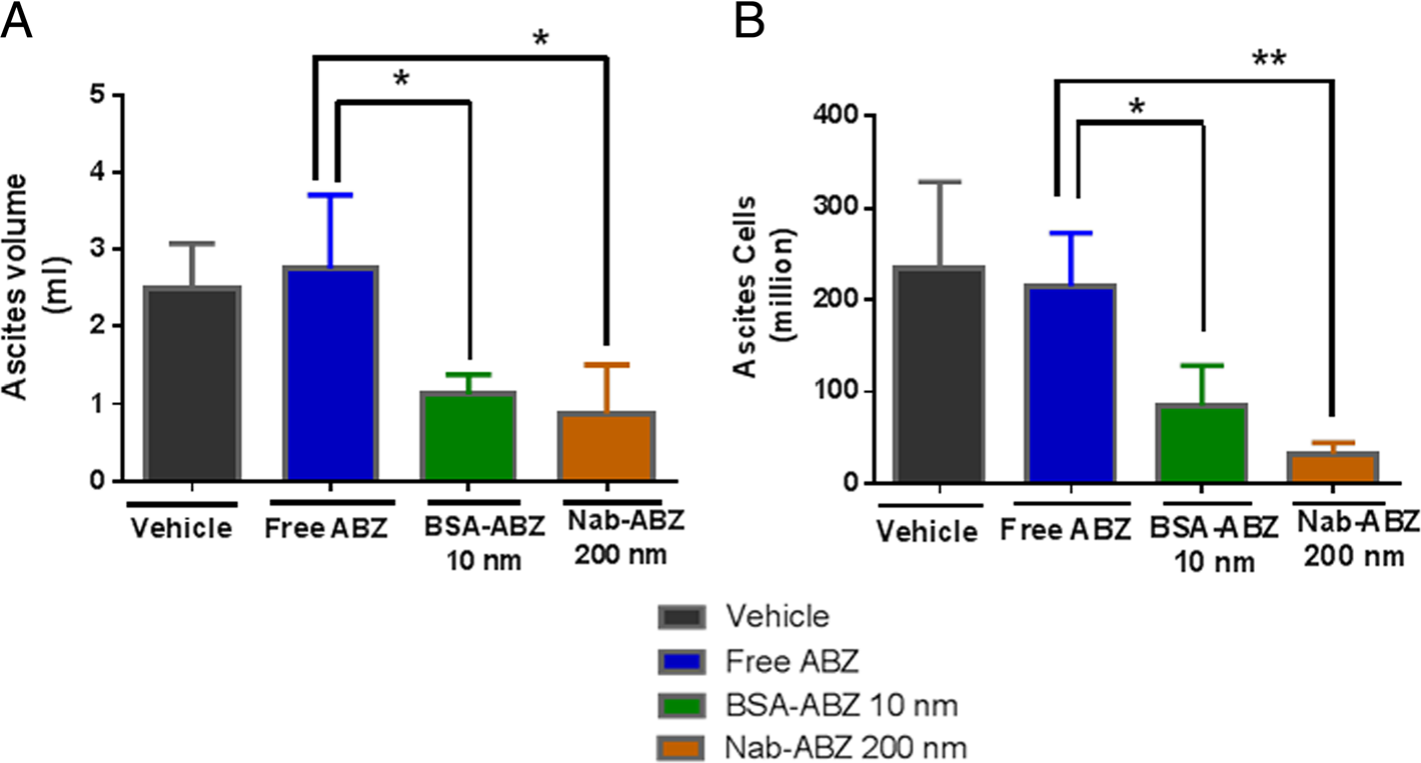
**ABZ nanoformulations reduced ascites fluid in peritoneal cavity of female nude mice.** Animals were received an IP injection of OVCAR3 cells in 4 treatment groups. All animals were sacrificed when the tumor controls displayed evidence of disease progression. **A)** Before sacrifice, the ascites volume was collected from abdominal cavity and measured. The ascites volume of animal group injected BSA-ABZ 10 nm and Nab-ABZ 200 nm were significantly lower (*P < 0.05) than free ABZ group. **B)** The ascites cells were washed with PBS and counted and the number of ascites cells were found to be significantly lower in BSA-ABZ 10 nm (*p < 0.05) and Nab-ABZ 200 nm (**p < 0.01) compare to free ABZ. Each column represents mean ascites fluid ± SD (n = 4).

Before euthanasia a peritoneal wash containing ascites fluid was collected from animals at the end of drug treatment and the volume was measured. The ascites volume of both groups was found to be significantly lower than the control group (free ABZ) (Figure 6A). The ascites cells were washed with PBS by centrifugation and counted and found to be significantly lower in the Nab-ABZ 200 nm group (Figure 6B). Remarkably, one mouse was detected to be free of ascites in the Nab-ABZ 200 nm group.

### ABZ nanoformulation suppresses VEGF in tumor and plasma of mice-bearing OVCAR3 tumor

**Figure 7:**
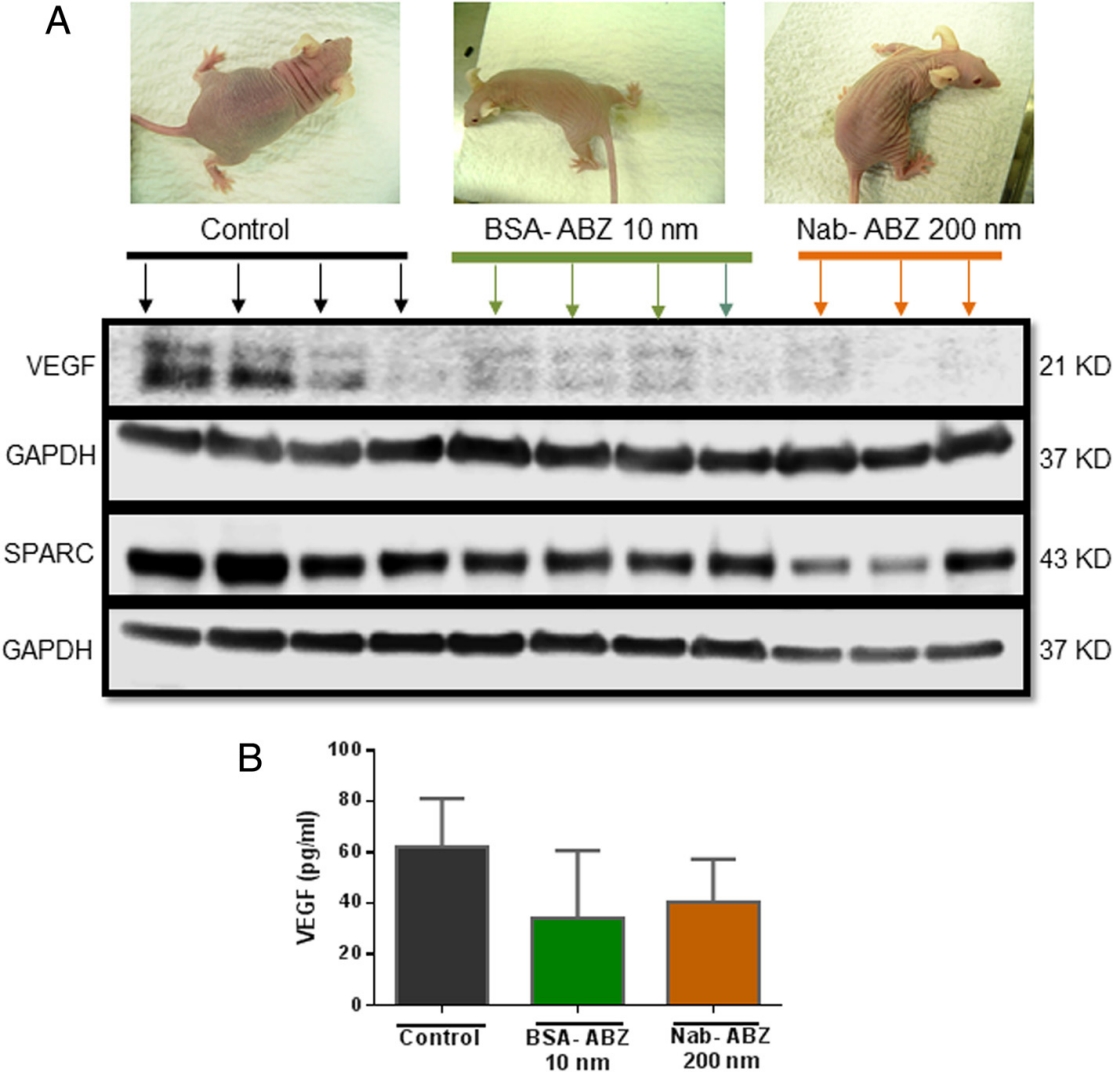
**The expression of VEGF and SPARC in tumor and VEGF in plasma. A)** The proteins were extracted from the tumor and concentration was determined. The expression of VEGF and SPARC was determined by western blot analysis. Tumor from each individual mouse was analysed separately for VEGF and SPARC levels. GAPDH was used as a control for equal protein loading. **B)** Following euthanasia, blood was collected by cardiac puncture and plasma samples were subjected to ELISA assay for VEGF levels. Each column represents mean VEGF levels ± SD (n = 4).

To further explore the changes at molecular level, tumor samples of the animals from each group were analysed for VEGF expression. Level of VEGF was reduced in both formulations (Figure 7A). SPARC is also correlated with the prognosis of cancer. SPARC expression was reduced in the Nab-ABZ 200 nm group. In contrast to the tumor VEGF, the VEGF level in plasma was not significantly reduced as shown in Figure 7B. The VEGF level was reduced by 45% in the BSA-ABZ 10 nm and 35% in the Nab-ABZ 200 nm compared to the control group.

## Discussion

The present study was designed to examine the effect of nanoparticle formulations on the disposition and activity of IP therapy in ovarian cancer xenograft model. A typical feature of epithelial ovarian carcinoma is its tendency to metastasize to the peritoneal cavity during disease progression. Poor drug penetration into the tumor tissues remains a significant challenge of conventional chemotherapy. Randomized phase III clinical trials in ovarian carcinoma showed increased overall survival with intraperitoneal chemotherapy as compared to intravenous chemotherapy [23]. Therefore, we attempted to examine the effect of intraperitoneal administration of nanoparticle formulations on tumor growth and ascites formation in ovarian cancer xenograft model. It has previously been reported that nanoparticles size < 50 nm are well absorbed in the peritoneum cavity [24] and easily pass through the lymph nodes to reach the thoracic lymph duct and the systemic circulation [25]. Therefore, one of the objectives of this study was to prepare small sized nanoparticles which may be well absorbed in the peritoneum cavity. In addition, these particles released ABZ slowly over an extended period of time to prevent the progression of metastasis intraperitoneally.

A previous study that focused on the development of cross-linked albumin nanoparticles for ABZ delivery, failed to show efficacy in *in-vivo* (Data not presented). In our study, we have modified the preparation method which also changed the hydrodynamic behaviour of particles as well as their sizes. *In-vitro* cytotoxicity assay revealed that the BSA-ABZ 10 nm was more toxic to both ovarian cancer cell lines (OVCAR3 and SKOV3) compared to the Nab-ABZ 200 nm and free ABZ and apparently nontoxic to the ovarian healthy cell (HOSE). The cellular internalization of alexa-488 conjugated albumin nanoparticles also demonstrated that both formulations were readily visible inside the cell membrane. However, BSA-ABZ 10 nm was more prominent than Nab-ABZ 200 nm (Figure 4D). FACS analysis also verified the internalization by quantification of the fluorescent intensity. BSA-ABZ 10 nm showed remarkably higher relative fluorescent intensity compared to the larger particles (Figure 4C). The increased cytotoxic effect of BSA-ABZ 10 nm is most likely due to the high ABZ concentration within the cell owing to higher cellular uptake efficiency. Similar to the *in-vitro* experiments, the increased antitumor efficacy of BSA-ABZ 10 nm in vivo may be due to high ABZ concentration in tumors mediated by albumin carrier.

Perhaps, the most important challenge addressed in this study was the delivery of BSA-ABZ 10 nm and Nab-ABZ 200 nm *in-vivo* which led to a superior anti-tumor activity. A literature search revealed that the *in-vivo* efficacy of nanoparticles depends on the uptake of nanoparticles by the tumor with the uptake being increased as particle size decreased [26,27]. Smaller particles lead to better uptake and therefore, better delivery of the drug [28] leading to higher toxicity. Nanoparticles experience more challenges *in vivo* because of the presence of variety of proteins and macromolecules that can degrade nanoparticles , be engulfed by phagocytic cells, or be taken away from the target site by the lymphatic system [29]. Therefore, the fate of nanoparticles in the *in vivo* setting depends on the degree and pattern of distribution of nanoparticles, stability, particle size and surface characteristics. In addition, nanoparticles of small particle diameter (<100 nm) and hydrophilic properties are capable of avoiding opsonisation and mononuclear phagocytic system (MPS) degradation resulting in enhanced blood circulation time [30,31].

Attempt to prepare suitable formulations as a free ABZ has been reported earlier. ABZ was previously observed to be well tolerated with a dose at 150 mg/kg, 3 times per week [11] and 450 mg/kg weekly with combination with chemotherapeutics [16] in mice model of ovarian cancer and 2500 mg/day in patients with ovarian cancer [32]. In the current study, BSA-ABZ 10 nm prevented tumor growth at ABZ concentration of only 0.5 mg/kg in mice model. The most interesting observation here is the higher efficacy in relation to a much lower concentration of ABZ. The intraperitoneal administration of BSA-ABZ 10 nm achieves better tumor control compared with Nab-ABZ 200 nm *in-vivo* although the concentration of ABZ was extremely low (10 μg/ml). Both formulations were capable of suppressing ascites volume and Nab-ABZ 200 nm significantly reduced ascites cells presented as floating cells in peritoneal wash. Expression of VEGF was down regulated in both formulations (Figure 7A). VEGF blockade has previously shown to inhibit ascites formation in several mouse model of ovarian cancer and the growth of solid tumors [33,34]. SPARC expression was significantly reduced in two mice in Nab-ABZ 200 nm group. Plasma VEGF concentration was also reduced in both groups (Figure 7B). In addition, the control mice developed visible tumor masses with massive ascites fluid, in contrast, mice treated with nanoparticle formulations showed a remarkable decrease in ascites production in both formulations (Figure 7A).

In fact, there are several possible explanations for the enhanced efficacy of BSA-ABZ 10 nm. Firstly, the albumin nanocarrier may improve the pharmacokinetic profile of ABZ and prolong its circulation time. These may result in higher accumulation in tumors due to enhanced permeation and retention (EPR) effects which allows nanoparticles to extravasate through the gaps between the endothelial cells and accumulate into the tumor tissues [35]. However, only NPs with specific size ranges can diffuse through the endothelium of tumor tissues and exploit the EPR effect [36]. The specific size of tumor vasculature defects depends on the cancer type, tumor site, and disease stage, but the gap is generally around few nm to 400 nm [37]. NPs must also be larger than 7 nm to avoid first-pass elimination in the kidney but smaller than 100 nm to avoid being cleared by the liver and spleen [38,39]*.* By exploiting albumin pathways, nanoparticles could transport across the endothelium of blood vessels via Gp60 and caveolae-mediated transcytosis process and also by association with the albumin-binding protein SPARC [19] which may play an important role in the increased tumor accumulation of albumin-bound drugs. Interestingly, SPARC is overexpressed in several aggressive cancers and downregulation of SPARC expression decreases cancer cell invasion and survival [40].

Secondly, since ABZ nanocarrier accumulates into the tumor, ABZ was released in a sustained fashion so that the tumor cells can be exposed to ABZ for a longer period of time. Thirdly, ABZ nanoparticles have relatively small sizes which may result in deeper penetration into tumor nodules. These findings highlight the importance of addressing several challenges in intraperitoneal drug delivery [25]. In addition, intraperitoneal administration has been regarded as a site specific delivery to peritoneal cavity that produces a higher local drug concentration. Possible mechanisms underlying the superior efficacy of BSA-ABZ 10 nm may be due to comparatively higher solubility and the ability to pass through the extracellular matrix into the tumor whereas Nab-ABZ 200 nm was probably more localised to the peritoneal cavity. Both of the formulations are capable of delivering drug constantly due to their sustained release profiles. As a result, released ABZ was retained in the interstitial space of the tumor for a longer period of time and exerted prolonged tumor abolishing effects in the peritoneal cavity [41].

## Conclusion

The current research shows satisfactory antitumor efficacy of albendazole since albumin nanocarrier was introduced in the formulation. The BSA-ABZ 10 nm is extremely effective to inhibit the tumor growth with low dose and Nab-ABZ 200 nm has a good indication for suppressing VEGF and ascites. However, both of these possibilities would open new areas of investigation for nanoparticle mediated drug delivery system but require further analysis of both formulations. Both approaches have significant benefits and potentials, but are limited by the access to the tumor which depends on the size of the particles. Hence, this novel delivery system for ABZ may provide a solution for the treatment of ovarian cancer with peritoneal ascites and overcome short comings such as adverse toxicities and chemo resistance that results from current treatment.

## Materials and methods

Albendazole, bovine serum albumin (BSA), nile red and Hydroxypropyl Methycellulose (HPMC) were purchased from Sigma Aldrich Ltd, Sydney, Australia. All reagents for cell culture were obtained from Invitrogen, Australia Pty Ltd. Alexa fluor-488 conjugated BSA was obtained from Invitrogen, USA. VEGF (sc-7269) mouse monoclonal IgG antibody, SPARC (sc-25574) rabbit polyclonal IgG antibody, Goat anti-mouse (sc-2031) was purchased from Santa Cruz Biotechnology. GAPDH (G8795) mouse monoclonal antibody was purchased from Sigma Aldrich Ltd, Sydney and anti-rabbit IgG # 7074 s was purchased from Cell Signalling.

### Preparation, characterization and drug release

Nanoparticle formulations were prepared by a modification of the method previously used for the preparation of cross-linked nanoparticles [42]. In brief, 50 mg (0.1% BSA) for 10 nm and 500 mg (1% BSA) for 200 nm was added to 50 ml of aqueous solution under constant stirring (600–800 rpm) at room temperature up to 30 minutes. After total dissolution, the solution was titrated to pH 8–9 with 1 N NaOH aqueous solution and stirred for 30 minutes. ABZ dissolved in THF and added in 0.1% BSA and 1% BSA at 10 μg/ml and 200 μg/ml respectively and stirred up to 4–5 hours to form nanoparticles between size range 8–10 nm and 200–250 nm. The residual THF was removed by heating up the solution up to 50–66°C in an oil bath with magnetic stirrer. Volume was measured and the concentration of ABZ was determined. Fluorescently labelled nanoparticles were prepared by using alexa-488 conjugated BSA for confocal study and nile red was loaded into albumin nanoparticles for FACS analysis. For animal study vehicle was prepared by making particles without drug [40]. Free ABZ was prepared as a control by dissolving ABZ in HPMC [43].

Albumin nanoparticles (0.5–1 mg/ml concentration) were solubilized in water to determine the particle size and size distribution by DLS (Malvern Instrument Ltd, South borough, MA, USA). The morphology of the nanoparticles was determined by TEM (CM 200, Philips, USA) using the protocol described previously [42]. Nanoparticle formulations were dissolved in PBS (pH 7.4) at a concentration of 10 μg / ml (10 nm) and 200 μg /ml (200 nm) and placed in an orbital shaker (C24 Edison, NJ, USA) at 100 rpm at 37°C for 192 hours (8 days). At predetermined time point, 500 μl PBS was collected from each sample and replaced with fresh PBS. Afterwards the samples were centrifuged at 12,000 rpm for 20 minutes and collected the supernatant containing free ABZ and mix with the same volume of methanol to dissolve ABZ. The concentration of free ABZ was measured in supernatant using HPLC (LC-20 Series, Shimadzu, Japan, Class VP software 7.4) and the release curve was then generated by plotting cumulative ABZ release versus time. The calculation was performed based on the equation generated from the calibration curve prepared from the release standard of ABZ in PBS and methanol.

### Cell culture

Human ovarian cancer cells (SKOV3, OVCAR3) and HOSE were purchased from American Type Culture Collection (ATTC, USA) and were maintained at 37°C in 5% CO2 in complete media using RPMI 1640 containing 10% (v/v) fetal bovine serum, streptomycin (100 μg/mL), and penicillin (100 units/mL). HOSE was cultured from ovarian epithelial cell medium supplemented with OEPICGS and penicillin streptomycin solution from ScienCell research laboratories, Carlsbad, CA, USA.

### *In-vitro* cytotoxicity

Cells were seeded into 96-well plates containing 3000 cells/ well and allowed to attach overnight. Once the cells reached 70% confluence, medium was replaced by serum free medium containing BSA-ABZ 10 nm, Nab-ABZ 200 nm and free ABZ (ABZ in ethanol) where ABZ concentration was 25, 50, 100, 200 and 500 ng/ml equivalent to 0.09, 0.19, 0.38, 0.75 and 1.88 μM respectively (the molecular weight of ABZ is 265.33 and BSA is 66.5 KDa). Equivalent ABZ doses were compared and cells were incubated with the nanoparticles for 72 hours. The cytotoxicity of ABZ was measured by previously established sulforhodamine B (SRB) assay [44]. The percentage proliferation of treatment wells was calculated as absorbance relative to control wells. The IC_50_ (the concentration required to decrease the cell number by 50%) was determined by using GraphPad Prism 6 software.

### Internalization of ABZ nanoformulation

SKOV3 cells were seeded into 6-well culture plates on cover slips and treated with BSA-ABZ 10 nm and Nab-ABZ 200 nm at a particle concentration 500 μg/ml for 2–3 hours. Medium was replaced with serum free RPMI media before treatment. The cells were washed 2 times with warm PBS and then fixed with ice cold 70% ethanol in 4°C for 15 minutes and again washed 3 times with cold PBS to remove ethanol and stained with propidium iodide (1 mg/ml) for 2 minutes to stain the nucleus. Again the cells were washed with PBS to remove all unattached staining. The slides were covered with permount ^R^ (oily gelatine) and closed with cover glasses and placed under the microscope. The nanoparticle distribution was imaged under a laser scanning microscope (Olympus Fluoview, FV 300, Japan) with a 60 X objective lens and the images were analysed using Olympus Fluoview 4.3 software.

For FACS analysis, SKOV3 cells were cultured in a small flask at a density of 0.8 million cells/flask. After overnight incubation, the culture medium was replaced with serum free medium and treated with albumin nanoparticles at a particle concentration of 500 μg/ml for 15, 30 and 60 minutes [45]. Fresh medium was used as a control. Cells were washed with PBS 3 times, trypsinized and centrifuged at 1500 rpm for FACS analysis using BD FACS Canto IIflow cytometer, USA and analysed the data using FlowJoV_10 software. The increase of fluorescence in the cells treated with nanoparticles relative to that in the untreated control cells expressed as the ratio of the intensity of cells incubated with particles and fluorescent intensity of untreated cells.

### Establishment of an ovarian cancer xenograft model

Female athymic nude mice (BALB/c), 8–10 weeks of age were purchased from the Animal Resources Centre (Perth, Australia). All animals were housed in a pathogen free environment according to the guideline of Animal Care and Ethics Committee of university of New South Wales (ACEC, UNSW) and all experiments were conducted according to the protocols approved by the committee. 16 female nude mice were used for this experiment (Ethic’s application ID 12/96A) in a project using St George animal house facility. Animals were received one week earlier before commencing the experiment.

The pre-cultured OVCAR3 cells were washed with sterile PBS 3 times, centrifuged at 1200 rpm at 4°C for 5 min to collect tumor cells and then immediately inoculated into the test animals. Each animal was injected intraperitoneally 7–10 million tumor cells suspended in PBS.

### Drug treatment and sample collection

One week after cell inoculation, mice were randomised into 4 groups of 4 mice each. Group 1 received vehicle, group 2, group 3 and group 4 were treated with free ABZ (50 mg/kg), BSA-ABZ 10 nm (0.5 mg/kg) and Nab-ABZ 200 nm (10 mg/kg) respectively. All groups received the drug intraperitoneally three times per week and the experiment continued up to 3 weeks. The ascites development was noted after 3 weeks and confirmed by comparison with controls. At the end of the experiment, the peritoneal cavity was washed with 2 ml of sterile normal saline and the peritoneal contents were mixed by kneading and then completely aspirated from each mouse [16]. Then the mice were sacrificed by an overdose of Lethabarb (Virbac, Australia) and the tumor weight and ascites volume was measured. The ascites was collected after each aspiration and was calculated by deducting 2 ml from the total volume collected. All visible tumor masses were carefully collected through laparotomy and the total mass weight was measured. Dissected tumors and ascites obtained from each group were all stored at – 80°C for subsequent analysis.

### Western blotting

To produce the lysate from tumor tissue, 100 mg tissue was lysed and homogenized in radio immuno-precipitation Assay (RIPA) buffer (Sigma, Australia) containing 10% protease inhibitor cocktail. The samples were then centrifuged at 10,000 g at 4° C for 10 minutes and the protein content in supernatant was quantified [17]. Fifty micrograms protein were fixed on 12% gels and electrophoresed for 2 hours at 85 V and transferred to Poly vinylidene Fluoride (PVDF) membranes. The membranes were incubated with the primary antibodies ( VEGF and SPARC, 1:200 dilutions, Santa Cruz Biotechnology) for overnight at 4°C followed by one hour incubation with secondary antibodies (anti-mouse from Santa Cruz Biotechnology and anti-rabbit from Cell Signalling, USA). The bands were visualized by an enhanced chemiluminescence detection kit (GE Healthcare, Australia). The blots were then stripped using Seppro western blot stripping buffer (Sigma, Australia) and re-probed with GAPDH (1:1000 dilution, Sigma, Australia).

### VEGF ELISA assay

The concentration of VEGF in mice plasma was determined using Human VEGF Quantikine Enzyme-Linked Immuno-Sorbent Assay (ELISA) Kit (R&D System) according to the manufacturer’s instructions.

### Statistical analysis

All statistical analyses were performed using the GraphPad Prism software package version 6.0 (GraphPad Software Inc., SanDiego, CA, USA). Data are presented as mean ± SD duplicate samples from two experiments. Differences between the groups were evaluated using Student’s t-test (unpaired) and two tailed p value was applied. P values (*p < 0.05) and (**p < 0.01) were considered to be statistically significant and very significant respectively.

## Additional file

Additional file 1:
**(A): Characterization of nab-ABZ 200 nm by dynamic light scattering (DLS) measurement; size distribution by number (top) and size distribution by volume (bottom).** Polydispersity index (PDI) of these NPs is 0.12. The result is average of 3 measurements. (B): Characterization of BSA-ABZ 10 nm by dynamic light scattering (DLS) measurement; size distribution by volume (top) and size distribution by number (bottom). Polydispersity index (PDI) of these NPs is 0.19. The result is average of 3 measurements.

## Abbreviations

ABZ: Albendazole
VEGF: Vascular endothelial growth factor
BSA-ABZ: Bovine Serum Albumin Albendazole
Nab-ABZ: Nano albumin formulation of albendazole
PBS: Phosphate buffer saline
SPARC: Secreted protein acidic and rich in cysteine
THF: Tetrahydrofuran
BSA: Bovine serum albumin
TEM: Transmission electron microscope
HOSE: Human ovarian surface epithelial
IP: Intraperitoneal
MPS: Mononuclear phagocytic system
DLS: Dynamic light scattering
EPR: Enhanced permeation and retention
ELISA: Enzyme-Linked Immuno-Sorbent Assay
