# Supplementary material for: Albumin nanoparticles increase the anticancer efficacy of albendazole in ovarian cancer xenograft model
Source: J Nanobiotechnology. 2015 Mar 25;13:25. doi: 10.1186/s12951-015-0082-8 (PMC4409778; doi:10.1186/s12951-015-0082-8)

Supplementary data

Nanoparticle characterization by DLS

Sample –Nab-ABZ

Particle size: 200-250 nm

PDI: 0.12


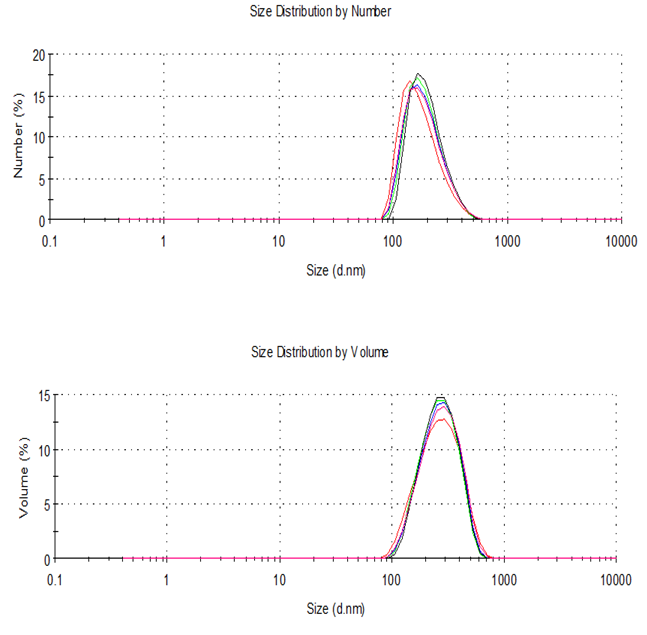


Nanoparticle characterization by DLS

Sample- BSA-ABZ

Particle size: 7 -10 nm

PDI: 0.19


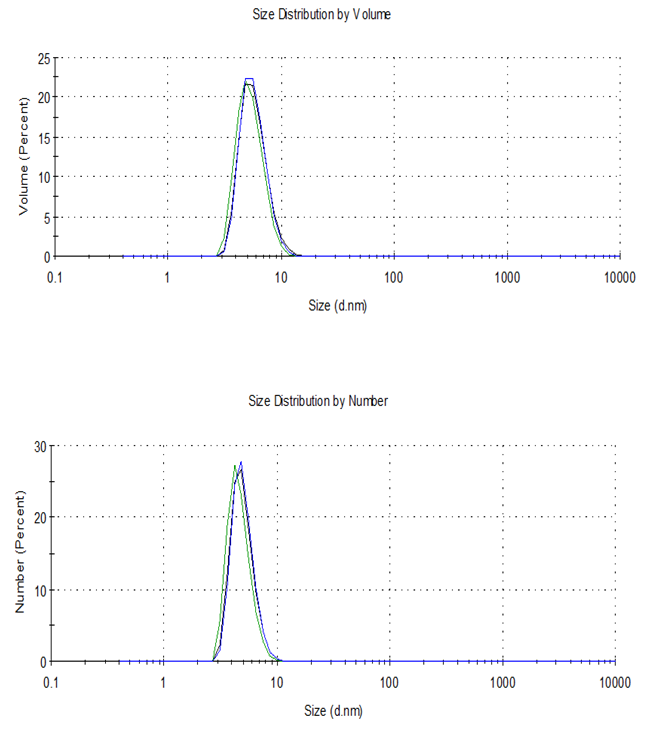

Supplement: Additional file 1: — (A): Characterization of nab-ABZ 200 nm by dynamic light scattering (DLS) measurement; size distribution by number (top) and size distribution by volume (bottom). Polydispersity index (PDI) of these NPs is 0.12. The result is average of 3 measurements. (B): Characterization of BSA-ABZ 10 nm by dynamic light scattering (DLS) measurement; size distribution by volume (top) and size distribution by number (bottom). Polydispersity index (PDI) of these NPs is 0.19. The result is average of 3 measurements. [file 12951_2015_82_MOESM1_ESM.docx]
